# Multi-view Performance Capture of Surface Details

**DOI:** 10.1007/s11263-016-0979-1

**Published:** 2017-01-21

**Authors:** Nadia Robertini, Dan Casas, Edilson De Aguiar, Christian Theobalt

**Affiliations:** 1 Max Planck Institute for Informatics, Saarbrücken, Germany; 2 CEUNES/UFES, São Mateus, Brazil; 3 Intel Visual Computing Insitute (Intel VCI), Saarbrücken, Germany

**Keywords:** Performance capture, Surface detail, Sums of Gaussian

## Abstract

This paper presents a novel approach to recover true fine surface detail of deforming meshes reconstructed from multi-view video. Template-based methods for performance capture usually produce a coarse-to-medium scale detail 4D surface reconstruction which does not contain the real high-frequency geometric detail present in the original video footage. Fine scale deformation is often incorporated in a second pass by using stereo constraints, features, or shading-based refinement. In this paper, we propose an alternative solution to this second stage by formulating dense dynamic surface reconstruction as a global optimization problem of the densely deforming surface. Our main contribution is an implicit representation of a deformable mesh that uses a set of Gaussian functions on the surface to represent the initial coarse mesh, and a set of Gaussians for the images to represent the original captured multi-view images. We effectively find the fine scale deformations for all mesh vertices, which maximize photo-temporal-consistency, by densely optimizing our model-to-image consistency energy on all vertex positions. Our formulation yields a smooth closed form energy with implicit occlusion handling and analytic derivatives. Furthermore, it does not require error-prone correspondence finding or discrete sampling of surface displacement values. We demonstrate our approach on a variety of datasets of human subjects wearing loose clothing and performing different motions. We qualitatively and quantitatively demonstrate that our technique successfully reproduces finer detail than the input baseline geometry.

**Electronic supplementary material:**

The online version of this article (doi:10.1007/s11263-016-0979-1) contains supplementary material, which is available to authorized users.

## Introduction

Over the last decade, performance capture techniques have enabled the 3D reconstruction of the motion and the appearance of real-world scenes, such as human motion or facial expressions, using a multi-camera capture setup (de Aguiar et al. 2008; Gall et al. 2009a; Bradley et al. 2008; Vlasic et al. 2008). Captured sequences are reconstructed using a space-time coherent 3D mesh, also known as 4D model, that reproduce the original scene or motion. This can be done, for example, by deforming a mesh or a rigged template such that it aligns with the images (de Aguiar et al. 2008; Vlasic et al. 2008), or by a per-frame independent reconstruction of the scene (Starck and Hilton 2007a) followed by a surface alignment step to convert the temporally inconsistent mesh geometries to a coherent model (Budd et al. 2013). However, many reconstructing methods only produce a coarse-to-medium scale 4D model that does not reproduce the high-frequency geometry detail present in the original scene. Finer scale shape detail can be then added in a second refinement step.

For fine surface detail reconstruction, some methods have used photo-consistency constraints via stereo-based refinement (de Aguiar et al. 2008; Starck and Hilton 2007b). However, such methods often turn to discrete sampling of local displacements, since formulating dense stereo based refinement as a continuous optimization problem has been more challenging (Kolev et al. 2009). Other methods for mesh refinement align the surface to a combination of silhouette constraints and sparse image features (Gall et al. 2009a). But such approaches merely recover medium scale detail and may suffer from erroneous feature correspondences between images and shape. Recently, shading-based techniques such as shape-from-shading or photometric stereo (Wu et al. 2011a, b; Vlasic et al. 2008) have been also proposed to capture small-scale displacements. However, these methods either are limited to be used in controlled and calibrated lighting setups, or they require a complex inverse estimation of lighting and appearance when they are applied in uncontrolled recording conditions.

This paper extends the work presented in Robertini et al. (2014) and it describes an effective solution to the refinement step using multi-view photo-consistency constraints. As input, our method expects synchronized and calibrated multiple video of a scene and a reconstructed coarse mesh animation, as it can be obtained with previous methods from the literature. Background subtraction or image silhouettes are not required for refinement.

Our first contribution is a new shape representation that models the mesh surface with a dense collection of 3D Gaussian functions centered at each vertex, each having an associated color, which we referred to as Surface Gaussians. A similar decomposition into 2D Gaussian functions, which we referred to as Image Gaussians, is applied to each input video frame. Leveraging this new Gaussian-based representation, our second contribution is a new formulation for dense photo-consistency-based surface refinement, which we formulate as a global optimization problem in the position of each vertex on the surface. Unlike previous performance capture methods, we are able to formulate the model-to-image photo-consistency energy that guides the deformation as a closed form expression, and we can compute its analytic derivatives. This also enables implicit handling of occlusions, as well as spatial and temporal coherence constraints, while preserving a smooth consistency energy function. We can effectively minimize this function in terms of dense local surface displacements with standard gradient-based solvers. In addition to these advantages, unlike many previous methods, our framework does not require a potentially error-prone sparse set of feature correspondences or discrete sampling and testing of surface displacements, and thus provides a new way of continuous optimization of the dense surface deformation.

We used our approach for reconstructing full-body performances of human actors wearing loose clothing, and performing different motions. Input coarse geometry of the scenes were obtained using a template-based method (Gall et al. 2009a; de Aguiar et al. 2008), or a template-free method (Starck and Hilton 2007b) followed by a surface alignment step (Budd et al. 2013). We demonstrate (Sect. 6) that our approach is able to reconstruct more of the correct fine-scale detail that is present in the input video sequences, than both of the baseline methods (i.e. template-based and template-free), for instance the wrinkles in a skirt. We also demonstrate these improvements quantitatively.

In contrast to the original work presented in Robertini et al. (2014), we improved our energy function by adding an additional temporal smoothing term to avoid jittering and artifacts in the final results. We provide a comprehensive description of all components of our energy function as well as the complete derivation of the analytic derivatives. We also added an extensive set of results and experiments to validate the performance of our improved approach.

## Related Work

Marker-less performance capture methods are able to reconstruct dense dynamic surface geometry of moving subjects from multi-view video, for instance of people in loose clothing, possibly along with pose parameters of an underlying kinematic skeleton (Theobalt et al. 2010). Most of them use data from dense multi-camera systems and are recorded under controlled studio environments. Some methods employ variants of shape-from-silhouette or active or passive stereo (Zitnick et al. 2004; Matusik et al. 2000; Starck and Hilton 2007b; Waschbüsch et al. 2005; Tung et al. 2009), which usually results in temporally incoherent reconstructions. A subsequent step, known as *surface tracking* or *surface alignment*, is used to convert the temporally incoherent geometry proxy to a coherent geometry that deforms over time to fit the reconstructed shapes. Cagniart et al. (2010) solve such free-form alignment sequentially by iterative close point matching of overlapping rigid-patches. Similarly, Budd et al. (2013) use a combination of geometric and photometric features in a non-sequential alignment framework. Volumentric constraints have also been used to formulate the surface tracking problem (Allain et al. 2015; de Aguiar et al. 2007). Nevertheless, regardless the accuracy of the method used for surface tracking, most of them result in a coarse-to-medium scale 4D model in which most of the high-frequency details are lost.

Space-time coherency is easier to achieve with model-based approaches that deform a static shape template (obtained by a laser scan or image-based reconstruction) such that it matches the subject, e.g. a person (Carranza et al. 2003; de Aguiar et al. 2008; Vlasic et al. 2008; Ballan and Cortelazzo 2008; Gall et al. 2009a) or a person’s apparel (Bradley et al. 2008). Some of them jointly track a skeleton and the non-rigidly deforming surface at the same time (Vlasic et al. 2008; Ballan and Cortelazzo 2008; Gall et al. 2009b); also multi-person reconstruction has been demonstrated (Liu et al. 2013). Other approaches use a generally deformable template without embedded skeleton to capture 4D models, e.g. an elastically deformable surface or volume (de Aguiar et al. 2008; Savoye 2013).

Most of the methods mentioned so far suffer from two main limitations: on one hand, the template-based methods that use an initial highly detailed scan model usually do not deform the fine-detail of the template according to the acquired per-frame imagery. This leads to incorrect surface detail on the final reconstructed meshes that does not match the captured dynamics (de Aguiar et al. 2008; Vlasic et al. 2008). On the other hand, methods that require the deformation of the per-frame reconstructed meshes to achieve temporal consistency (Starck and Hilton 2007b; Budd et al. 2013; Cagniart et al. 2010) tend to output a coarse reconstruction of the sequence. In both cases true fine detail needs to be recovered in a subsequent step. Medium scale non-rigid 4D surface detail can be estimated by using a combination of silhouette constraints and sparse feature correspondences (Gall et al. 2009a). Other approaches use stereo-based photo-consistency constraints in addition to silhouettes to achieve denser estimates of finer scale deformations (Starck and Hilton 2007b; de Aguiar et al. 2008). It is an involved problem to phrase dense stereo-based surface refinement as a continuous optimization problem, as it is done in variational approaches (Kolev et al. 2009). Thus, stereo-based refinement in performance capture often resorts to discrete surface displacement sampling which are less efficient, and with which globally smooth and coherent solutions are harder to achieve. Alternatively, fine-detail surface deformation caused by garment wrinkling has been investigated using cloth-specific approaches. Popa et al. (2009) enhance reconstructed surfaces using a wrinkle generation method based on the recorded shadows.

Fine-scale detail can be also recovered using active lighting methods, e.g. shape-from-shading or photometric stereo (Wu et al. 2011a). Many of these approaches require controlled and calibrated lighting (Hernandez et al. 2007; Vlasic et al. 2009a; Ahmed et al. 2008; Vlasic et al. 2009b), which reduces their applicability in real-world scenarios. For example, Hernandez et al. (2007) use red, green and blue lights from different directions to estimate surface normals with a photometric stereo approach. This enables detailed reconstruction of cloth, even untextured. Ahmed et al. (2008) estimate surface reflectance and time-varying normal fields on a coarse template mesh to incorporate garment details such as wrinkles and folds. Vlasic et al. (2009b) reproduce high-resolution geometric detail by capturing multi-view normal maps in a large lighting dome that provides a series of novel spherical lighting configurations. More recently, shading-based refinement of dynamic scenes captured under more general lighting was shown Wu et al. (2011b), but these approaches are computationally challenging as they require to solve an inverse rendering problem to obtain estimates of illumination, appearance and shape at the same time.

Our problem formulation is inspired by the work of Stoll et al. (2011) who used 2D and 3D Gaussian functions for marker-less skeletal pose estimation. The use of implicit surfaces for shape and motion estimation from multi-view video was originally proposed by Plankers and Fua (2003), who use smooth implicit surfaces attached to an articulated skeleton to approximate a human 3D shape. This representation allows to define a distance function of data points and models that is differentiable. Similarly, Ilic and Fua (2006) create *implicit meshes* by attaching triangular primitives to the faces of explicit 3D meshes. Surface reconstruction and refinement is performed by optimizing the implicit mesh (exploiting its attractive properties) and propagating the deformation to the explicit mesh for rendering.

Our technique has some similarity to the work of Sand et al. (2003) who capture skin deformation as a displacement field on a template mesh. However, they require marker-based skeleton capture, and only fit the surface to match the silhouettes in multi-view video. This paper extends the formulation of stereo-based surface refinement as a continuous optimization problem described in Robertini et al. (2014), which is based on a new surface representation with Gaussian functions, with an additional temporal smoothing term that improves the final results avoiding jittering and artifacts.

**Fig. 1:**
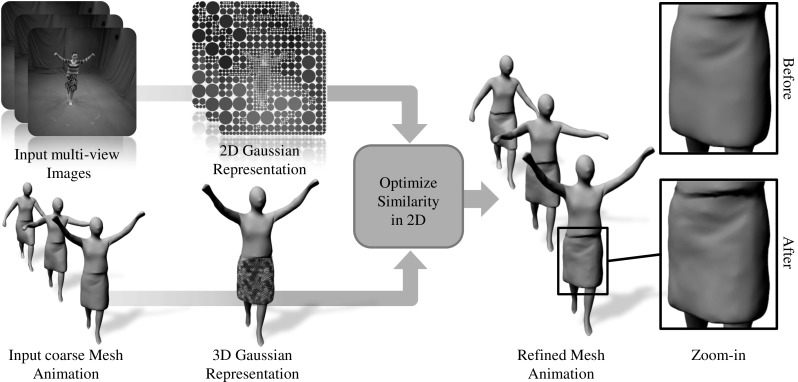
Our approach takes as input a coarse topologically-consistent mesh animation and a set of input images from a multi-view calibrated and synchronized camera setup. Both the input mesh and image sequences are converted to an implicit representation resembling respectively a collection of 3D Surface Gaussians and 2D Image Gaussians. Then, the color consistency between the two representations is optimized in 2D for each camera view, resulting in a refined mesh animation with additional captured surface details (Color figure online)

## Overview

Figure 1 shows an overview of our approach. The input to our system is a calibrated and synchronized multi-view video sequence showing images of the human performer and a spatio-temporally coherent set of coarse meshes, reconstructed from related approaches (Gall et al. 2009a; Starck and Hilton 2007b; de Aguiar et al. 2008; Budd et al. 2013).

Our algorithm refines the initial set of coarse geometries by incorporating fine dynamic surface details to the meshes. First, an implicit representation of the input mesh using a dense collection of 3D Gaussian functions, referred to as Surface Gaussians, with associated colors is created. The input images are also represented as a set of 2D Gaussian Functions, referred to as Image Gaussians, associated to image patches in each camera view. Thereafter, continuous optimization is performed to maximize the color consistency between the collection of Surface and Image Gaussians. The optimization displaces the Surface Gaussians along the associated vertex normal of the coarse mesh which yields the necessary vertex displacement. Our optimization method has a smooth energy function, that can be expressed in closed form and it allows us to analytically compute derivatives, enabling the possibility of using efficient gradient-based solvers.

## Implicit Model

Our framework converts the input coarse animation and the input multi-view images into implicit representations using a collection of Gaussians: Surface Gaussians (i.e. 3D Gaussian functions) on the mesh surface with associated colors and Image Gaussians (i.e. 2D Gaussian functions), with associated colors, assigned to image patches in each camera view.

### Surface Gaussians

**Fig. 2:**
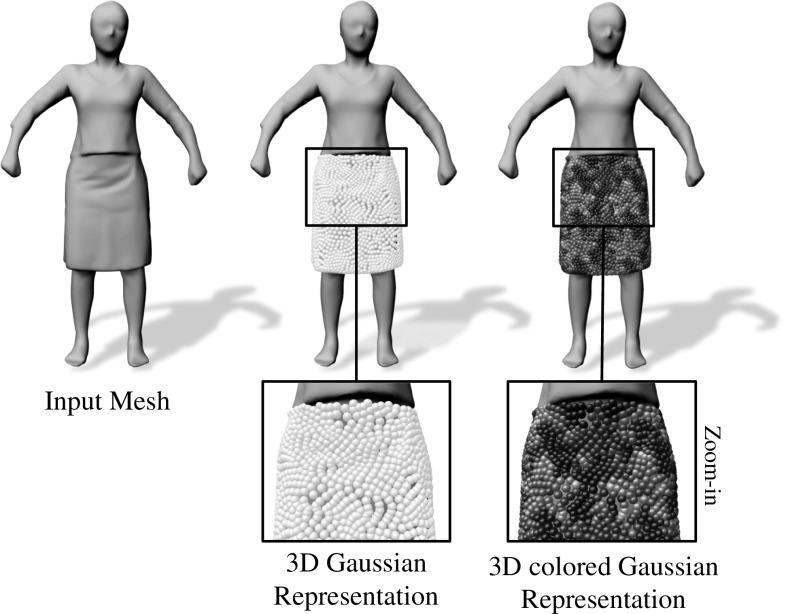
Surface Gaussian initialization pipeline. Starting from the input mesh (*left*), we populate the regions in which we want to recover the fine geometric detail (in this case, the skirt) with 3D Gaussians (*center*). Finally, a color is assigned to each 3D Gaussian based on the underlying reprojected pixel average from the best-camera view (*right*)

Our implicit model for the input mesh is obtained by placing a Surface Gaussian at each mesh vertex $v_s$, $\forall s \in \{0\dots n_s-1\}$, $n_s$ being the number of vertices. An isotropic Gaussian function on the surface is defined simply with a mean $\hat{\mu }_s$, that coincides with the vertex location, and a standard deviation $\hat{\sigma }_s$ as follows:

1 $$\begin{aligned} \hat{G}_s(\hat{x}) = \frac{1}{\sqrt{\sigma _s \sqrt{\pi }}} exp\left( -\frac{||\hat{x}-\hat{\mu }_s||^2}{2 \hat{\sigma }_s^2}\right) , \end{aligned}$$

with $\hat{x} \in \mathbb {R}^3$. Note that although $\hat{G}_s(\hat{x})$ has infinite support, for visualization purposes we represent it as a sphere centered at $\hat{\mu }_s$ with $\hat{\sigma }_s$ $mm$ radius (see Fig. 2). In contrast to the unnormalized representation presented in Robertini et al. (2014), we normalize the Surface Gaussians with normalization factor of $\frac{1}{\sqrt{\sigma _s \sqrt{\pi }}}$. In Sect. 5 we mathematically validate this choice and show quantitative improvements on the overall energy formulation.

We further assign a HSV color value $\eta _s$ to each Surface Gaussian. In order to derive the colors we choose a reference frame where the initial coarse reconstruction is as close as possible to the real shape. This is typically the first frame in each sequence. For each vertex $v_s$ of the input mesh, we chose the most direct camera, i.e. where vertex normal and camera viewing direction align best. The Surface Gaussian associated to $v_s$ is projected to the image from the best camera view and the corresponding pixel color average is assigned as a color attribute.

### Image Gaussians

Our implicit model for the input images *I*(*c*) of all cameras $c \in \{0\dots n_c-1\}$, $n_c$ being the number of cameras, is obtained by assigning Gaussian functions $G_i(x)$, $x \in \mathbb {R}^2$, which we referred to as Image Gaussians, to each image patch of all camera views. Similar to Stoll et al. (2011) we decompose each input frame into squared regions of coherent color by means of quad-tree decomposition. We set a maximum quad-tree depth $T_{qt}$ and recursively split the image domain of each camera view in smaller regions. Then, we fuse together neighboring patches of similar color up to a color similarity threshold $T_{fuse}$. The color similarity between two regions having colors $\eta _1$ and $\eta _2$ is computed by simply taking the Euclidean distance $||\eta _1 - \eta _2||^2$.

**Fig. 3:**
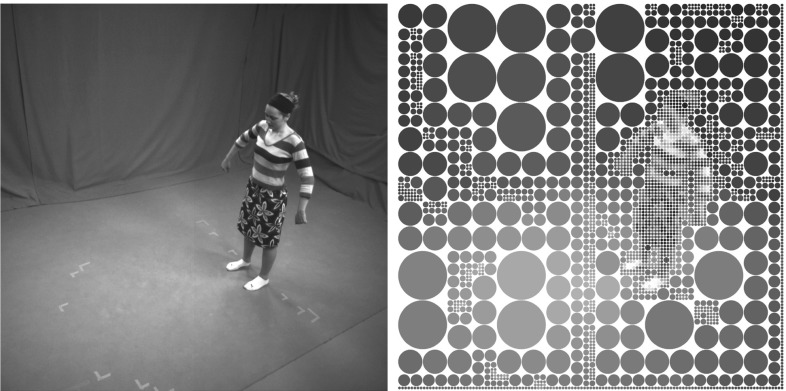
The input image (*left*) and the estimated collection of Image Gaussians (*right*), here represented by circles. The Image Gaussians are assigned to patches of coherent color in the input image and the underlying average pixel color is assigned as an additional attribute

After this bottom-up approach is completed, an Image Gaussian is assigned to each patch (Fig. 3), such that its mean $\mu _i \in \mathbb {R}^2$ corresponds to the patch center, and its standard deviation $\sigma _i$ to half of the square patch side length. The underlying average HSV color $\eta _i$ is also assigned as an additional attribute.

### Projection of Surface Gaussians

Our surface refinement approach, described later in detail in Sect. 5, requires the evaluation of the similarity between Surface Gaussians $\hat{G}_s$ and Image Gaussians $G_i$. In order to ease such evaluation, Surface Gaussians $\hat{G}_s$ are projected to the 2D image space of each camera view by using the corresponding camera projection matrix *P*. Specifically, a Surface Gaussian mean $\hat{\mu }_s$ is projected to a camera image frame as follows:

2 $$\begin{aligned} \mu _{s} = \left( \begin{array}{c} \frac{[P \hat{\mu }_s^h]_x}{[P \hat{\mu }_s^h]_z} \\ \frac{[P \hat{\mu }_s^h]_y}{[P \hat{\mu }_s^h]_z} \end{array} \right) = \left( \begin{array}{c} \frac{[\mu _s^p]_x}{[\mu _s^p]_z} \\ \frac{[\mu _s^p]_y}{[\mu _s^p]_z} \end{array} \right) \in \mathbb {R}^2, \end{aligned}$$

where $[{\hat{\mu }}_s^h]_{x,y,z}$ are the respective coordinates of the mean in homogeneous coordinates (i.e. the 4*th* dimension is set to 1), $[{\mu }_s^p]_{x,y,z}$ are the respective coordinates of the projected mean in homogeneous coordinates, and $\mu _s$ is the projected mean. Similarly, the standard deviation $\hat{\sigma }_s$ of a Surface Gaussian is projected using the following formula

3 $$\begin{aligned} \sigma _s = \frac{\hat{\sigma }_s f}{[P \hat{\mu }_s^h]_z}= \frac{\hat{\sigma }_s f}{[\mu _s^p]_z} \in \mathbb {R}, \end{aligned}$$

where $f$ is the camera focal length and ${\sigma }_s$ the projected standard deviation.

## Surface Refinement

We employ an analysis-by-synthesis approach to refine the input coarse mesh animation, at every frame, by optimizing the following energy $\mathbf E (M)$ with respect to the collection of Surface Gaussian means $M = \{\hat{\mu }_0,\dots \hat{\mu }_{n_s-1}\}$:

4 $$\begin{aligned} \mathbf E (M) = E_{sim} - w_{reg} E_{reg} - w_{temp} E_{temp}. \end{aligned}$$

The term $E_{sim}$ measures the color similarity of the projected Surface Gaussians with the Image Gaussians obtained from each camera view. $E_{reg}$ is used to keep the distribution of the Surface Gaussians geometrically smooth, whereas $w_{reg}$ is an user defined smoothness weight. The additional term $E_{temp}$ is used to temporally smooth the displacements of the Surface Gaussians over time to avoid visual artifacts such as jittering, whereas $w_{temp}$ is a user defined weight.

We constrain the Surface Gaussians to only move along the corresponding vertex (normalized) normal direction $N_s$:

5 $$\begin{aligned} \hat{\mu }_{s} = \hat{\mu }_{s}^{init} + N_s k_s \in \mathbb {R}^3 \end{aligned}$$

where $\hat{\mu }_s^{init}$ is the initial Surface Gaussian mean inizialized as the vertex position $v_s$ at the beginning of each frame, and $k_s$ is the unknown vertex displacement.

This hard constraint brings two main advantages: first, it forces the Surface Gaussians to maintain a regular distribution on the surface, and secondly it highly reduces the number of parameters to optimize for (single scalar displacements $k_s$, instead of the 3 dimensions $[\hat{\mu }_s]_x, [\hat{\mu }_s]_y$ and $[\hat{\mu }_s]_z$), resulting in higher performances as well as better-posed convergence.

By maximizing $\mathbf {E}(M)$ for each frame in terms of the collection of Surface Gaussian means *M*, we aim at best surface-image similarity with the best distribution in space (on the surface) and time (across frames). We define each term of $\mathbf {E}(M)$ analytically and compute its derivatives with respect to the unknown displacements $k_s, \forall s \in \{0\dots n_s-1\}$, which we then set to 0 for maximization purposes. The derivatives are:

6 $$\begin{aligned} \begin{aligned} \frac{\partial \mathbf E }{\partial k_s}&= \frac{\partial }{\partial k_s}\left( E_{sim} - w_{reg} E_{reg} - w_{temp} E_{temp}\right) \\&= \frac{\partial E_{sim}}{\partial k_s} - w_{reg} \frac{\partial E_{reg}}{\partial k_s} - w_{temp} \frac{\partial E_{temp}}{\partial k_s} \\ \end{aligned} \end{aligned}$$

In the next sections, we describe each term in detail and provide the full derivation of the analytic derivatives.

### Similarity Term

We exploit the power of the implicit Gaussian representation of both input images and surface in order to derive a closed-form analytical formulation for our similarity term. In principle, a pair of Image Gaussian and projected Surface Gaussian should have high similarity measures when they show similar properties in terms of color and their spacial localization is sufficiently close. This measure can be formulated as the integral of the product of the projected Surface Gaussian $G_s(x)$ and Image Gaussian $G_i(x)$, weighted by their color similarity $T(\delta _{i,s})$, as follows:

7 $$\begin{aligned} {\varPhi }_{i,s} = T_{{\varDelta }_c}(\delta _{i,s}) \left[ \int _{{\varOmega }}{G_i(x) G_s(x) \partial {x}}\right] ^2 \end{aligned}$$

In the above equation $\delta _{i,s} = || \eta _i - \eta _s ||^2 \in \mathbb {R}^{+}$ measures the Euclidean distance between the colors, ${\varDelta }_c$ is the maximum color distance allowed (after which the color similarity should drop to 0), and $T_{{\varDelta }}(\delta ): \mathbb {R} \rightarrow \mathbb {R}$ is the *Wendland* radial basis function (Wendland 1995) modeled by:

8 $$\begin{aligned} T_{{\varDelta }}(\delta ) = \left\{ \begin{array}{l l} \Big (1 - \frac{\delta }{{\varDelta }}\Big )^4 \Big (4 \frac{\delta }{{\varDelta }} + 1\Big ) &{} \quad \text {if }\delta < {\varDelta }\\ &{}\\ 0 &{} \quad \text {otherwise} \end{array} \right. \end{aligned}$$

Applying $T_{{\varDelta }}$ function on $\delta$ results in a smooth color similarity measure that is equal 1 if $\delta = 0$, i.e.  $T_{{\varDelta }}(0) = 1$ and smoothly decreases towards 0 as $\delta$ approaches ${\varDelta }$, i.e.  $\lim _{\delta \rightarrow {\varDelta }} T_{{\varDelta }}(\delta ) = 0$.

The main advantage of using a Gaussian representation is that the integral in Eq. 7 has a closed-form solution, namely another Gaussian with combined properties:

9 $$\begin{aligned} {\varPhi }_{i,s}= & {} T_{{\varDelta }_c}(\delta _{i,s}) \left[ \int _{{\varOmega }} \frac{1}{\sqrt{\pi \sigma _s \sigma _i}} exp\left( -\frac{1}{2}\frac{||x-\mu _{i}||^2}{\sigma _i^2}\right) \right. \nonumber \\&\left. \times ~exp\left( -\frac{1}{2}\frac{||x-\mu _{s}||^2}{\sigma _s^2}\right) \partial x \right] ^2\nonumber \\= & {} T_{{\varDelta }_c}(\delta _{i,s}) \left[ \frac{\sqrt{2 \sigma _s \sigma _i}}{\sqrt{(\sigma _{s}^2+\sigma _{i}^2)}} exp\left( -\frac{1}{2}\frac{||\mu _i - \mu _s ||^2}{\sigma _{s}^2+\sigma _{i}^2}\right) \right] ^2\nonumber \\= & {} T_{{\varDelta }_c}(\delta _{i,s}) 2 \frac{\sigma _{s} \sigma _{i}}{\sigma _{s}^2+\sigma _{i}^2} exp\left( -\frac{||\mu _i - \mu _s ||^2}{\sigma _{s}^2+\sigma _{i}^2}\right) \end{aligned}$$

**Fig. 4:**
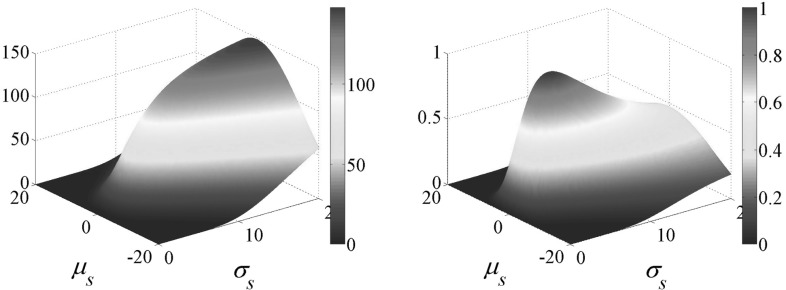
Similarity $E_{sim}$ evaluated for a Surface Gaussian with varying mean $\mu _s$ and standard deviation $\sigma _s$ against a fixed Image Gaussian having $\sigma _i = 5$ and $\mu _i = 0$. *Left* the energy obtained without Gaussian normalization as in Robertini et al. (2014). *Right* the energy obtained by normalizing the Gaussians as explained in this paper. Both plots have a maxima in $\mu _s = 0 = \mu _i$, however only the normalized energy on the right has *max* $E_{sim} = 1$ for $\sigma _s = 5 = \sigma _i$, while the un-normalized plot has $\lim _{\sigma _s\rightarrow \infty } E_{sim} = \infty$

The use of normalized Surface Gaussians with the chosen normalization factor allows to mathematically constrain the overlap ${\varPhi }_{i,s}$ in the interval [0, 1], which has appealing properties concerning the next formulations’ steps. Although we do not make use of this, it is worth mention that ${\varPhi }_{i,s}$ with normalized Gaussian also eases the optimization of the size (i.e. standard deviation) along with the mean of the Surface Gaussians, that was previously impractical (see Fig. 4 for comparison).

To compute $E_{sim}$, we first calculate the overlap of the set of Surface Gaussian against the set of Image Gaussians for each camera view, obtained by summing-up all overlaps ${\varPhi }_{i,s}$, $\forall i, s$. Then, we normalize the result considering the number of cameras $n_c$ and the maximum obtainable overlap, which can be easily found counting out the Image Gaussians $\sum _i {\varPhi }_{i,i} = \sum _i 1 = n_i^c$, $\forall c$:

10 $$\begin{aligned} E_{sim} = \frac{1}{n_{c}} \sum _{c=0}^{n_{c}-1} \left[ \frac{1}{n_i^c} \sum _{i = 0}^{n_i^c - 1} min \left( \sum _{s = 0}^{n_s-1} {\varPhi }_{i,s}, 1\right) \right] \end{aligned}$$

such that $E_{sim} \in [0,1]$. The use of normalized Gaussians contributes in an improvement in performance ($3\%$ w.r.t. the unnormalized version). In this equation, the inner minimization implicitly handles occlusions on the surface as it prevents occluded Gaussian projections into the same image location to contribute multiple times to the energy. This is an elegant way for handling occlusion while preserving at the same time energy smoothness.

**Derivative for** $E_{sim}$: In order to calculate the derivative of $E_{sim}$, we note that most of its terms are constant with respect to $k_s$, except the projected means $\mu _{s}$ and the variances $\sigma _{s}$, within the term ${\varPhi }_{i,s}$.

Using homogeneous coordinates, expressed throughout the paper using the superindex *h*, we first compute the Surface Gaussian mean in 2D image space, $\mu _{s}^h$, by projecting the constrained Surface Gaussian mean $\hat{\mu }_s^h$ from Eq. 5, using the camera projection matrix $P \in \mathbb {R}^{4 \times 4}$:

11 $$\begin{aligned} \mu _{s}^{h} = P \hat{\mu }_s^h = P(\hat{\mu }_s^{init} + N_s^h k_s) \in \mathbb {R}^3 \end{aligned}$$

where $\hat{\mu }_s^{init}$ is the initial Surface Gaussian mean, initialized as the vertex position $v_s$, in homogeneous coordinates. The derivative of $\mu _{s}^{h}$ with respect to $k_s$ is defined as:

12 $$\begin{aligned} \frac{\partial \mu _{s}^{h}}{\partial k_s}= & {} \frac{\partial }{\partial k_s}(P(\hat{\mu }_s^{init} + N_s^h k_s)) = P\frac{\partial }{\partial k_s}(\hat{\mu }_s^{init} + N_s^h k_s) \nonumber \\= & {} P \left( 0 + N_s^h \frac{\partial }{\partial k_s}(k_s)\right) = P N_s^h \end{aligned}$$

Combining Eqs. 2 and 12, the derivative of $\mu _s$ evaluates to:

13 $$\begin{aligned} \begin{aligned}&\frac{\partial \mu _{s}}{\partial k_s} = \left( \begin{array}{c} \frac{\partial }{\partial k_s}\left( \frac{{[\mu _{s}^{h}]}_x}{{[\mu _{s}^{h}]}_z} \right) \\ \frac{\partial }{\partial k_s}\left( \frac{{[\mu _{s}^{h}]}_y}{{[\mu _{s}^{h}]}_z} \right) \end{array} \right) = \left( \begin{array}{c} \frac{\frac{\partial {[\mu _{s}^{h}]}_x}{\partial k_s} {[\mu _{s}^{h}]}_z- {[\mu _{s}^{h}]}_x\frac{\partial {[\mu _{s}^{h}]}_z}{\partial k_s}}{{{[\mu _{s}^{h}]}_z}^2} \\ \frac{\frac{\partial {[\mu _{s}^{h}]}_y}{\partial k_s} {[\mu _{s}^{h}]}_z- {[\mu _{s}^{h}]}_y\frac{\partial {[\mu _{s}^{h}]}_z}{\partial k_s}}{{{[\mu _{s}^{h}]}_z}^2} \\ \end{array} \right) \\&= \left( \begin{array}{c} \frac{\partial }{\partial k_s}\left( {[\mu _{s}^{h}]}_x\right) - [\mu _s]_x \frac{\partial }{\partial k_s}\left( {[\mu _{s}^{h}]}_z\right) \\ \frac{\partial }{\partial k_s}\left( {[\mu _{s}^{h}]}_y\right) - [\mu _s]_y \frac{\partial }{\partial k_s}\left( {[\mu _{s}^{h}]}_z\right) \\ \end{array} \right) \frac{1}{{[\mu _{s}^{h}]}_z} \\&= \left( \begin{array}{c} {{[P N_s^h]}_x- [\mu _s]_x {[P N_s^h]}_z}\\ {{[P N_s^h]}_y- [\mu _s]_y {[P N_s^h]}_z}\\ \end{array} \right) \frac{1}{[P (\hat{\mu }_s^{init} + N_s^h k_s)]_z}. \end{aligned} \end{aligned}$$

The derivative with respect to $k_s$ of the projected variance $\sigma _s$ is calculated by applying simple derivation rules:

14 $$\begin{aligned} \begin{aligned} \frac{\partial \sigma _{s}}{\partial k_s}&= \frac{\partial \sigma _{s}}{\partial {[\mu _{s}^{h}]}_z} \frac{\partial {[\mu _{s}^{h}]}_z}{\partial k_s} = \frac{-f \hat{\sigma }_s}{({[\mu _{s}^{h}]}_z)^2}\frac{\partial {[\mu _{s}^{h}]}_z}{\partial k_s} \\&= \frac{-\sigma _{s}}{{[\mu _{s}^{h}]}_z}\frac{\partial {[\mu _{s}^{h}]}_z}{\partial k_s} = \frac{-\sigma _{s}}{{[\mu _{s}^{h}]}_z}{[P N_s^h]}_z\in \mathbb {R}, \end{aligned} \end{aligned}$$

Therefore, the derivative of the term ${\varPhi }_{i,s}$ with respect to $k_s$ is obtained by substituting Eqs. 13 and 14 in 9, which generates:

15 $$\begin{aligned} \begin{aligned}&\frac{\partial }{\partial k_s}({\varPhi }_{i,s}) = T_{{\varDelta }_c}(\delta _{i,s}) 2 \frac{\partial }{\partial k_s}\Bigg (\frac{\sigma _{s}\sigma _{i}}{\sigma _{s}^2+\sigma _{i}^2} e^{-\frac{||\mu _i - \mu _s ||^2}{\sigma _{s}^2+\sigma _{i}^2}}\Bigg )\\&= T_{{\varDelta }_c}(\delta _{i,s}) 2 \Bigg \{2 \frac{\sigma _{s}\sigma _{i}}{\sigma _{s}^2+\sigma _{i}^2} e^{-\frac{||\mu _i - \mu _s ||^2}{\sigma _{s}^2+\sigma _{i}^2}}\Bigg [\frac{\partial {[\mu _{s}^{h}]}_z}{\partial k_s} \Bigg (-\frac{1}{2} +\\&+ \frac{\sigma _{s}^2}{\sigma _{s}^2+\sigma _{i}^2} - \frac{||\mu _i - \mu _s ||^2\sigma _{s}^2}{(\sigma _{s}^2+\sigma _{i}^2)^2 }\Bigg )\frac{1}{{[\mu _{s}^{h}]}_z} + \frac{(\mu _i - \mu _s) \frac{\partial \mu _{s}}{\partial k_s}}{\sigma _{s}^2+\sigma _{i}^2}\Bigg ]\Bigg \}\\&= T_{{\varDelta }_c}(\delta _{i,s}) 4 \frac{\sigma _{s}\sigma _{i}}{\sigma _{s}^2+\sigma _{i}^2} e^{-\frac{||\mu _i - \mu _s ||^2}{\sigma _{s}^2+\sigma _{i}^2}}\Bigg [{[P N_s^h]}_z\Bigg (-\frac{1}{2} +\\&+ \frac{\sigma _{s}^2}{\sigma _{s}^2+\sigma _{i}^2} - \frac{||\mu _i - \mu _s ||^2\sigma _{s}^2}{(\sigma _{s}^2+\sigma _{i}^2)^2 }\Bigg )\frac{1}{{[\mu _{s}^{h}]}_z} + \frac{(\mu _i - \mu _s) \frac{\partial \mu _s}{\partial k_s}}{\sigma _{s}^2+\sigma _{i}^2}\Bigg ]\end{aligned} \end{aligned}$$

Finally, the derivative of $E_{sim}$ with respect to $k_s$ is:

16 $$\begin{aligned} \frac{\partial E_{sim}}{\partial k_s} = \frac{1}{n_{c}} \sum _{c=0}^{n_{c}-1} \frac{1}{n_i^c} \sum _{i = 0}^{n_i^c-1} \left\{ \begin{array}{l l} \frac{\partial {\varPhi }_{i,s}}{\partial k_s} &{} \text {if} \sum \nolimits _{s = 0}^{n_s-1} {\varPhi }_{i,s} < 1\\ &{}\\ 0 &{} \text {otherwise} \end{array} \right. \end{aligned}$$

### Regularization Term

The regularization term constraints the Surface Gaussians in the local neighborhood such that the final reconstructed surface is sufficiently smooth. This is accomplished by constraining the displacements $k_s$ along the normals by minimizing the following equation:

17 $$\begin{aligned} E_{reg} = \sum _{s = 0}^{n_s-1} \frac{1}{|{\varPsi }(s)|}{\sum _{j \in {\varPsi }(s)} T_{{\varDelta }_d}(\delta _{s,j}) \left( k_s - k_j\right) ^2}, \end{aligned}$$

where ${\varPsi }(s)$ is a set of Surface Gaussian indices that are neighbors of $G_s$, $T_{{\varDelta }}(\delta )$ is defined in Eq. 8, $\delta _{s,j} \in \mathbb {R}^{+}$ is the geodesic surface distance between $G_s$ and $G_j$ measured in number of edges and ${\varDelta }_d$ is the maximum allowed geodesic distance (after which $T_{{\varDelta }_d}$ drops to 0). Since we assume fixed surface topology for our experiments, $\delta _{sj}$ does not change, and in particular is constant with respect to the degrees of freedom $k_s$. We compute the geodesic distance among all vertices and all possible neighbors only once for each sequence. The effect of the minimization of $E_{reg}$ is to maintain a smooth surface where all close neighbors show similar displacements the more they are close to each other. A similar formulation in the case of free motion of the Surface Gaussian without any normal constraints would be harder to formulate. It would possible require more complex and additional terms to guarantee smooth and regular surface distribution of the resulting vertex positions.

**Table 1:** User-defined parameters of the energy function $\mathbf E$, together with their description, values interval and default value

Parameter	Description	Values int.	Def.
$w_{reg}$	Regularization weight	[0, 1]	$5e^{-7}$
$w_{temp}$	Temporal weight	[0, 1]	$1e^{-7}$
$\sigma _s$	Standard deviation, $\forall G_s$	$(0,\infty )$, [mm]	5
$T_{qt}$	Quad-tree depth threshold	$[0,log_2(I^{W,H}) ]$	9
$T_{fuse}$	Color similarity for fusion threshold	$[0,\infty )$	0.05
$T_{color}$	Color similarity threshold	$[0,\infty )$	0.15
$T_{dist}$	Threshold on pixel distance	$[0,\infty )$, [*px*]	30
${\varDelta }_d$	Max geodesic distance	$[0,\infty )$, $[\# edges]$	2

The quad-tree depth threshold $T_{qt}$ and color similarity threshold $T_{fuse}$ are used to subdivide the input 2D images as described in Sect. 4.2 and find the Image Gaussians $G_i$. $T_{qt}$ can be at most the logarithm in base 2 of the minimum among the image width $I^W$ and the image height $I^H$, expressed as $log_2(I^{W,H})$. The thresholds on color $T_{color}$ and pixel distance $T_{dist}$ between Surface $G_s$ and Image Gaussians $G_i$ define for each $G_s$ the subset of $G_i$ against which the similarity function is evaluated, excluding less contributing Image Gaussians (see Sect. 5.4)

**Derivative for** $E_{reg}$: The derivative of $E_{reg}$ with respect to $k_s$ is calculated by simple derivation rules as follows:

18 $$\begin{aligned} \begin{aligned}&\frac{\partial E_{reg}}{\partial k_s}\! = \!\frac{\partial }{\partial k_s}\! \left( \sum _{s = 0}^{n_s-1} \frac{1}{|{\varPsi }(s)|}{\sum _{j \in {\varPsi }(s)} \! T_{{\varDelta }_d}(\delta _{s,j}) \! \left( k_s\! - \!k_j\right) ^2}\!\right) \\&\quad = \frac{1}{|{\varPsi }(s)|} \sum _{j \in {\varPsi }(s)} \! T_{{\varDelta }_d}(\delta _{sj}) \left( \frac{\partial \left( \! k_s \! - \! k_j \! \right) ^2 }{\partial k_s} + \frac{\partial \left( \! k_j \! - k_s \! \right) ^2}{\partial k_s}\right) \\&\quad = \frac{1}{|{\varPsi }(s)|} \sum _{j \in {\varPsi }(s)} \! T_{{\varDelta }_d}(\delta _{s,j}) \left( 2 \left( k_s\! - k_j\right) - 2 \left( k_j\! - k_s\right) \right) \\&\quad = \frac{4}{|{\varPsi }(s)|} \sum _{j \in {\varPsi }(s)} T_{{\varDelta }_d}(\delta _{s,j}) \left( k_s - k_j\right) \end{aligned} \end{aligned}$$

### Temporal Smoothing Term

The temporal smoothing term is used to constraint the displacements $k_s$ over time, generating a smooth temporal deformation and avoiding jitter and artifacts. This additional term is defined as follows:

19 $$\begin{aligned} E_{temp} = \sum _{s=0}^{n_s - 1} \left( \frac{1}{2} (k_s^{f-2} + k_s^{f}) - k_s^{f-1} \right) ^2\end{aligned}$$

where $k_s^{f-2}$, $k_s^{f-1}$ and $k_s^{f}$ are respectively the normal displacement $k_s$ computed 2 frames before, 1 frame before and at the current frame. This formulation is inspired by the acceleration law, aiming at obtaining time consistent results with smooth acceleration. The smoothing term comes into play after computing the displacements for the first 2 frames, when the constants for the first frame $k_s^{1}$, and second frame $k_s^{2}$ are known.

**Derivative for** $E_{temp}$: The derivative of $E_{temp}$ with respect to $k_s^f$ at the current frame *f* is calculated by simple derivation rules as follows:

20 $$\begin{aligned} \frac{\partial E_{temp}}{\partial k_s}= & {} 2 \left( \frac{1}{2} \left( k_s^{f-2} + k_s^{f}\right) \! -\! k_s^{f-1} \! \right) \! \left( \frac{1}{2} (0 + 1)\! -\! 0\right) \nonumber \\= & {} \frac{1}{2} (k_s^{f-2} + k_s^{f}) - k_s^{f-1}\end{aligned}$$

### Optimization

Our energy function $\mathbf {E}$ can be efficiently optimized using an iterative gradient-based approach. For each iteration *t* of the maximization process, we compute the derivative of $\mathbf {E}^t$ with respect to each $k_s, s \in \{0\dots n_s - 1\}$, as obtained summing-up all energy term derivatives together, following Eq. 6.

To improve computational efficiency, we evaluate the overlap ${\varPhi }_{i,s}$ only for visible Surface Gaussians from each camera view. Explicit visibility computation is performed only once at the beginning of each frame, by considering each Surface Gaussian as simple vertices. The implicit occlusion handling takes care of consistently handling new occlusions that might arise during optimization. The Gaussian overlap is then computed against visible projected Surface Gaussians and Image Gaussians in a local neighborhood, by considering only the closest Image Gaussians up to a distance threshold $T_{dist}$ in number of pixels and a color distance threshold $T_{color}$. Table 1 summarizes the main user defined parameters as well as their default values.

We efficiently optimize our energy function $\mathbf E$ using a *conditioned gradient ascent* approach. The general gradient ascent method is a first-order optimization procedure that aims at finding local maxima by taking steps proportional to the energy gradient. It uses a scalar factor, the conditioner $\gamma$, associated to the analytical derivatives that increases (resp. decreases) step-by-step when the gradient sign is constant (resp. fluctuating).

**Fig. 5:**
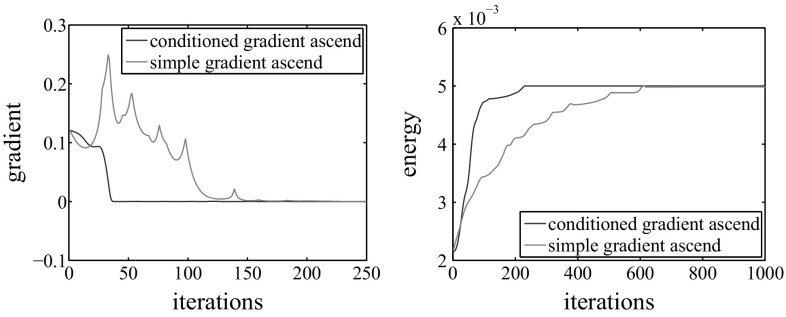
Comparison between conditioned gradient ascent (*red*) used in this paper, and simple gradient ascent (*green*) optimization. *Left* gradient intensity $\overline{\nabla }(\mathbf {E})_s$ per iteration of a single parameter $k_s$. *Right* the energy $\mathbf E$ per iteration. The conditioned gradient ascent has faster convergence to the local maxima while keeping a smooth gradient curve (Color figure online)

We define the gradient at the iteration *t* of the maximization operation as ${\nabla }(\mathbf {E})^t= \frac{\partial }{\partial k_s}(\mathbf E )^t$ and proceed as follows. At each optimization step *t* we update the displacements $k_s^t$ based on the current normalized gradient $\overline{\nabla }(\mathbf {E})^t$ and conditioner $\gamma ^t$

21 $$\begin{aligned} k_s^t = k_s^{t-1} + \overline{\nabla }(\mathbf {E})^t \gamma ^t \end{aligned}$$

where $k_s^{0} = 0$, $\forall s = 0\dots n_s-1$, and $\overline{\nabla }(\mathbf {E})^t$ is the normalized gradient computed considering the maximum ${\nabla }(\mathbf {E})^t$ among all $s = 0\dots n_s -1$ at the current step to ensure values in the interval [0, 1]:

22 $$\begin{aligned} \overline{\nabla }(\mathbf {E})^t = \frac{{\nabla }(\mathbf {E})^t}{max\left( {\nabla }(\mathbf {E})^t, s = 0\dots n_s-1\right) }\end{aligned}$$

The conditioner is initially set to $\gamma ^0 = 0.1$, then we update it based on the gradients at previous and current step as follows:

23 $$\begin{aligned} \gamma ^{t+1} = \left\{ \begin{array}{l l} min\left( 1.2 \gamma ^{t},\frac{{\varDelta }_{\gamma }}{\overline{\nabla }(\mathbf {E})^t}\right) &{} \quad \text {if }\left( \overline{\nabla }(\mathbf {E})^{t-1} \overline{\nabla }(\mathbf {E})^{t}\right) > 0\\ &{}\\ 0.5 \gamma ^{t} &{} \quad \text {otherwise} \end{array} \right. \end{aligned}$$

where ${\varDelta }_{\gamma } = 1$ mm is the maximum step size. We additionally check if the gradient has dramatically decreased in magnitude, and if so further dampen the conditioner based on the gradient ratio:

24 $$\begin{aligned} \gamma ^{t+1} = 0.25 \frac{\overline{\nabla }(\mathbf {E})^{t-1}}{\overline{\nabla }(\mathbf {E})^{t}} \gamma ^t \end{aligned}$$

The use of the conditioner brings three main advantages: it allows for faster convergence to the final solution, it prevents undesired zig-zag-ing while approaching local maxima, and it constraints at the same time the analytical derivative size. Such benefits are depicted in Fig. 5, which shows the impact of the conditioner on the convergence curve trend. For each frame, we perform at least 5 and at most 1000 iterations, and stop when $\frac{|\mathbf E ^{t} - \mathbf E ^{t-1}|}{max(1,\mathbf E ^{t},\mathbf E ^{t-1})} \le 1e^{-8}$.

Once the convergence has been reached (typically around iteration 200 for all sequences, see Fig. 5), we update the vertex positions of the input mesh at the current frame by simply displacing them along the corresponding normal using the found optimal $k_s$. Note that in practice, when rendering the final resulting mesh sequence, we add an extra $\epsilon$ to the computed vertex displacement $k_s$. This is needed to compensate for the small surface bias (shrink along the normal during optimization) that is due to the spatial extent of the Gaussians. Hence, we update the vertex position

25 $$\begin{aligned} v_s = v_s^{init} + N_s \cdot (k_s + \epsilon ) \end{aligned}$$

where $v_s^{init}$ is the original location of the vertex, $N_s$ is the corresponding unchanged normal and $\epsilon = \sigma _s$ throughout this work.

## Results

In this Section we describe our datasets and provide detailed qualitative and quantitative evaluation of the results, as well as complexity and time performances of our approach. Finally, we discuss the main directions for a future work.

### Datasets

We tested our approach on five different datasets: *skirt*, *dance*, *pop2lock*, *handstand* and *wheel*. Input multi-view video sequences, as well as camera settings and initial coarse mesh reconstruction were provided by Gall et al. (2009a) and Starck and Hilton (2007b). All sequences are recorded with 8 synchronized and calibrated cameras and number of frames ranging between 120 and 640 (see Table 2). Input coarse meshes are obtained using techniques based on sparse feature matching, shape-from-silhouette and multi-view 3D reconstruction, and therefore lack of surface details.

**Table 2:** Details for each sequence

Sequence	*Skirt*		*Dance*		*Handstand*	*Wheel*	*Pop2lock*		*Synthetic*
Published by	Gall et al. (2009a)						Starck and Hilton (2007b)		Ours
Cameras	8								10
Frames	640		574		150	120	250		1
Frame rate (Hz)	40								n/a
Camera type	Phase space vision camera								Simulated
Image resolution (px)	1004 $\times$ 1004						$1920\times 1080$		$1280\times 720$
Vertices	12095		6002		13809	13809	10006		42
Used/total cameras	7/8								10/10
3D Gaussians	12095		3430		12095	12095	3880		42
2D Gaussians per frame	$\approx$9000		$\approx$9800		$\approx$15200	$\approx$15200	$\approx$13000		Variable
Ground truth	n/a, Flow displacement + silhouette error								Constructed
Input meshes	Orig	Smooth	Orig	Smooth	Orig	Original	Orig	Smooth	Orig
Timing (min/frame)	$\approx$20		$\approx$3		$\approx$30	$\approx$30	$\approx$3		$\approx$0.04

This table summarizes the main settings for all the used sequences, as well as optimization-related settings, e.g. approximate amount of 3D and 2D Gaussians per frame

In order to refine the input mesh sequences, we first subdivide the input coarse topology by inserting additional triangles and vertices. We then generate a collection of Gaussians on the surface, as described in Sect. 3. Since most of the fine-scale deformations happen on the clothing, we focus the refinement on those areas, generating Surface Gaussians only for the corresponding vertices. Table 2 shows the amount of Surface Gaussians created for each sequence.

To visually enhance the capabilities of our refinement approach in capturing fine-scale surface details, we have created 3 additional datasets by smoothing the input meshes of the sequences *dance*, *skirt* and *pop2lock*. By doing so we eliminated most of the baked-in surface details, and use the over-smoothed mesh animations as input to our system.

We additionally tested our method on a *synthetic* sequence, consisting on a 42-vertices sphere model with randomly generated colors. We generate three different scenarios: the unchanged sphere, the sphere with randomly displaced vertices along the corresponding vertex normals, and the sphere with randomly displaced vertices without any normal constraint. We then render each of the scenarios from 10 arbitrary view-points, which we use as the input multi-view images, and we use each scenario geometry as a ground truth. We run our approach in each scenario, using the unchanged geometry as input coarse mesh, and the rendered views as input multi-view images. Finally we compare the output of each scenario with the ground truth geometry. In this Section we show results of such experiment.

### Computational Complexity and Efficiency

The complexity of our approach for each frame *f* and camera *c* depends on the number of iterations $n_t$ performed by the gradient-based solver, as well as the number of Image Gaussians $n_i^c$ and Surface Gaussians $n_s$, $O(n_t n_i^c n_s)$. These 3 quantities variate depending on the sequence, frame and specific camera. The order of complexity is obtained considering the maximum among the possible values for each sequence. For each frame, at most $n_t = 1000$ iterations are performed (see Sect. 5.4), and at most each single pixel of each camera view is taken as Image Gaussian, resulting in $n_i^c = 1004\times 1004$ for the sequences from Gall et al. (2009a) and $n_i^c = 1920\times 1080$ for Starck and Hilton (2007b) (see Table 2). However, convergence to a solution is typically reached in less iterations. The number of Image Gaussians is also smaller than the number of image pixels for each camera, as for each Surface Gaussian we only take into account the closest Image Gaussians in terms of spacial and color distance, as described in Sect. 5.4. It can be easily shown that our approach has linear complexity with respect to varying Surface Gaussians.

We evaluated the performance of our system on an Intel Xeon Processor E5-1620, Quad-core with Hyperthreading and 16 GB of RAM. Table 2 summarizes the performances we obtained for the 6 tested sequences. The computation time can be further reduced by parallelizing orthogonal optimization steps, such as the evaluation of the overlap ${\varPhi }_{i,s}, \forall i,s$ (see Sect. 5.1). Our algorithm is particularly suited for implementation on GPU, which we believe has a strong impact on the time performances.

### Evaluation

**Fig. 6:**
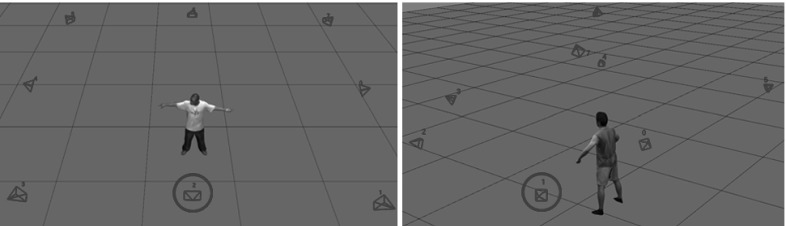
Camera set-up for the *pop*2*lock* (*left*) and *dance* (*right*) sequence. All the remaining sequences, have similar camera settings as in *dance*. The circled camera is the excluded one from the optimization, which is used for evaluation

Our results, shown in Figs. 10 and 11 together with the accompanying video, demonstrate that our approach is able to plausibly reconstruct more correct fine-scale details, e.g. the wrinkles and folds in the skirt, and it produces closer model alignment to the images than the baseline methods (Gall et al. 2009a; Starck and Hilton 2007b). Most of the shown sequences do not provide ground-truth data, i.e. all sequences except the synthetic sequence. To quantitatively evaluate our approach on the sequences for which no ground-truth geometry is available, we computed the optical flow error. To this end, we first textured the input and resulting mesh models by assigning Surface Gaussians colors to the corresponding vertices. Then, we used the classical variational approach by Brox et al. (2004) to generate displacement flow vectors between the input images of a single camera view and the reprojected textured mesh models for each frame and pixel. Then, for each frame, we compute the average displacement error, by dividing the sum of the displacements norm by the number of pixels. The evaluation is performed on a camera view that is excluded from the optimization (see Fig. 6 for a visualization of the camera setup).

**Fig. 7:**
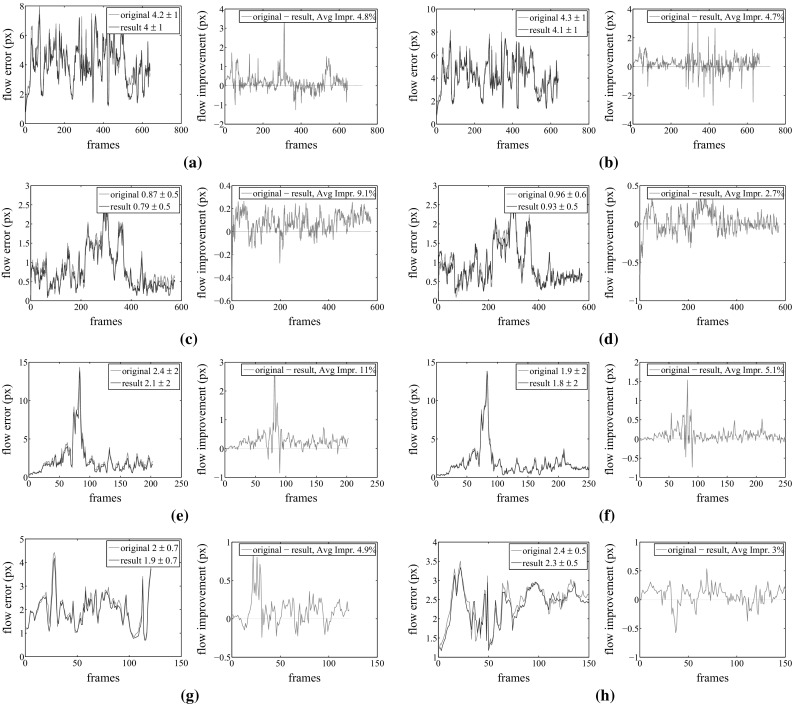
Average flow displacement error of the refined meshes, for 5 different datasets, including over-smoothed version. In each subfigure: (*left*) normalized flow error per frame for original, in *red*, and refined, in *blue*, mesh sequences. (*Right*) Difference between input and resulting flow error. Notice how the resulting error is consistently decreased across all sequences. We register about $2.7{-}11$% average improvement of the sequence quality with respect to the input flow error. See the text for detailed evaluation. **a** skirt, **b** smooth skirt, **c** dance, **d** smooth dance, **e** pop2lock, **f** smooth pop2lock, **g** wheel, **h** handstand (Color figure online)

As shown by the graphs in Fig. 7 our method significantly decreases the average flow displacement error, leading to quantitatively more accurate results (up to $11\%$ improvement). High percentage of flow error reduction between input and refined meshes is also obtained in the purposely smoothed datasets. This confirms that our approach successfully recovers true surface detail even when using very coarse geometry.

Our approach is able to capture the deformation dynamics happening on the clothing in all sequences, as well as fine-scale details, especially for highly textured regions, e.g. the *skirt* sequence. Details on the inner plain-colored regions, e.g. shirt folds in the *dance* sequence, are hard to reproduce with our approach, since they lack of sufficiently distinctive color information in the Surface Gaussians neighborhood.

**Fig. 8:**
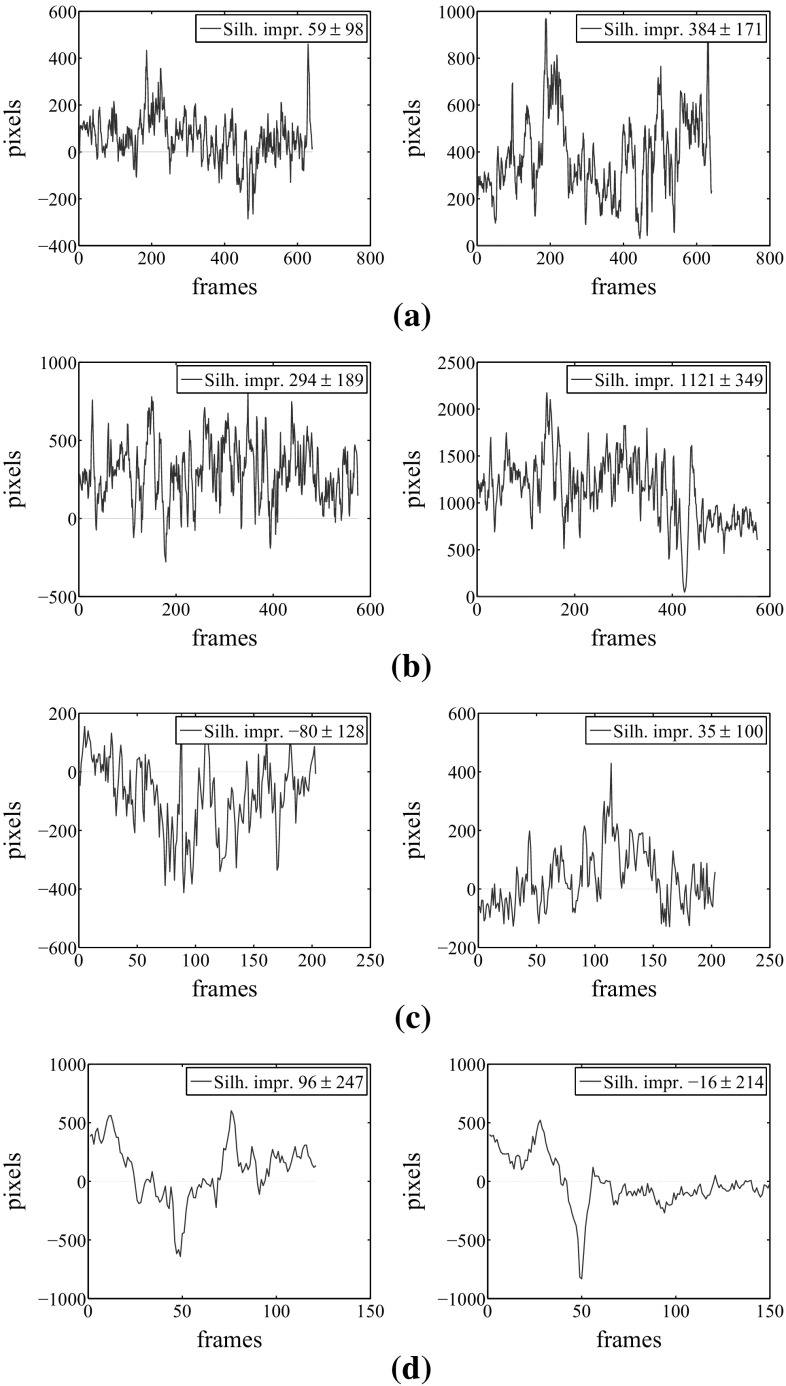
Silhouette improvement of the refined meshes for 5 different datasets, including over-smoothed sequences. In each subfigure: pixel improvement with respect to the specified input sequence per frame. See the text for detailed evaluation. Manually estimated true cloth pixels to be used as a reference for the silhouette pixel improvement are stated in the captions of each sequence. **a** skirt, smooth skirt, Cloth px $\approx 16$ K, **b** dance, smooth dance, Cloth px $\approx 35$ K, **c** pop2lock, smooth pop2lock, Cloth px $\approx 19$ K, **d** wheel, handstand, Cloth px $\approx 30$ K

**Fig. 9:**
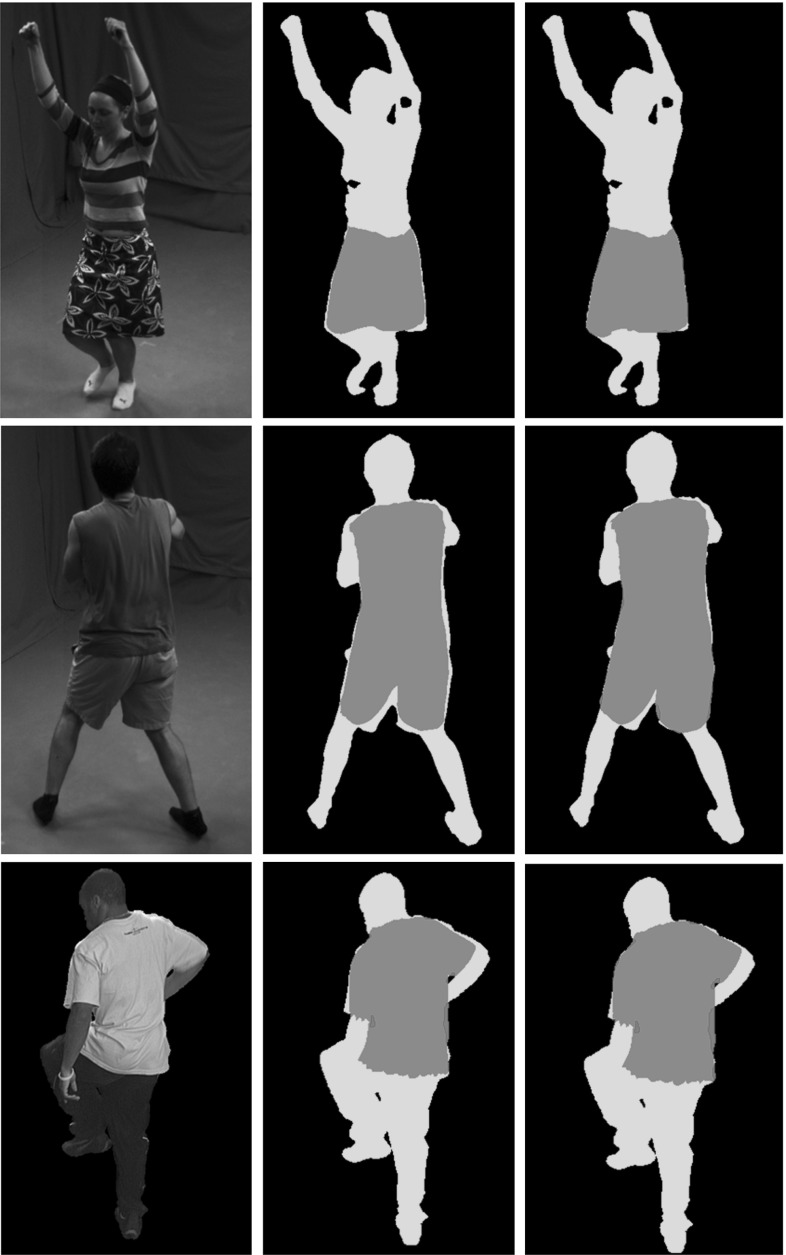
Silhouette overlap evaluation of the refined meshes shown for *skirt*, *dance* and *pop*2*lock* sequences. From *left to right*: input image, silhouette overlap of the input and refined mesh from an unused view. We used the following color code for the silhouette overlap: *Purple* true positives, *Green* false negatives, *Red* false positives (Color figure online)

Results demonstrate that our approach also improves the alignment of the reconstructed meshes with respect to the input image, without explicitly relying on any precomputed foreground segmentation. As opposite to most of the silhouette-based approaches (Starck and Hilton 2007a), our optimization approach smoothly and continuously improves the alignment to the silhouettes, without using error-prone correspondence finding and discrete optimization. We show silhouette reprojection improvement for all tested sequences in Figs. 8 and 9. The silhouette improvement per frame is computed by counting the false positive and false negative (i.e. wrong) pixels in the original and refined sequences, obtained by subtracting the ground-truth silhouette image pixels with the reprojected mesh pixels from an unused camera. Then, we take the difference between original and refined false pixels, which implicitly resembles the amount of newly found correct pixels. Notice, that our computations are performed using the available full-body silhouette images, although we just refine the clothing parts. We manually counted the average amount of true pixels in the clothing areas for each sequence as a reference, see the Figure caption 8. Follows detailed evaluation of each tested sequence.

#### *Skirt* Sequence

**Fig. 10:**
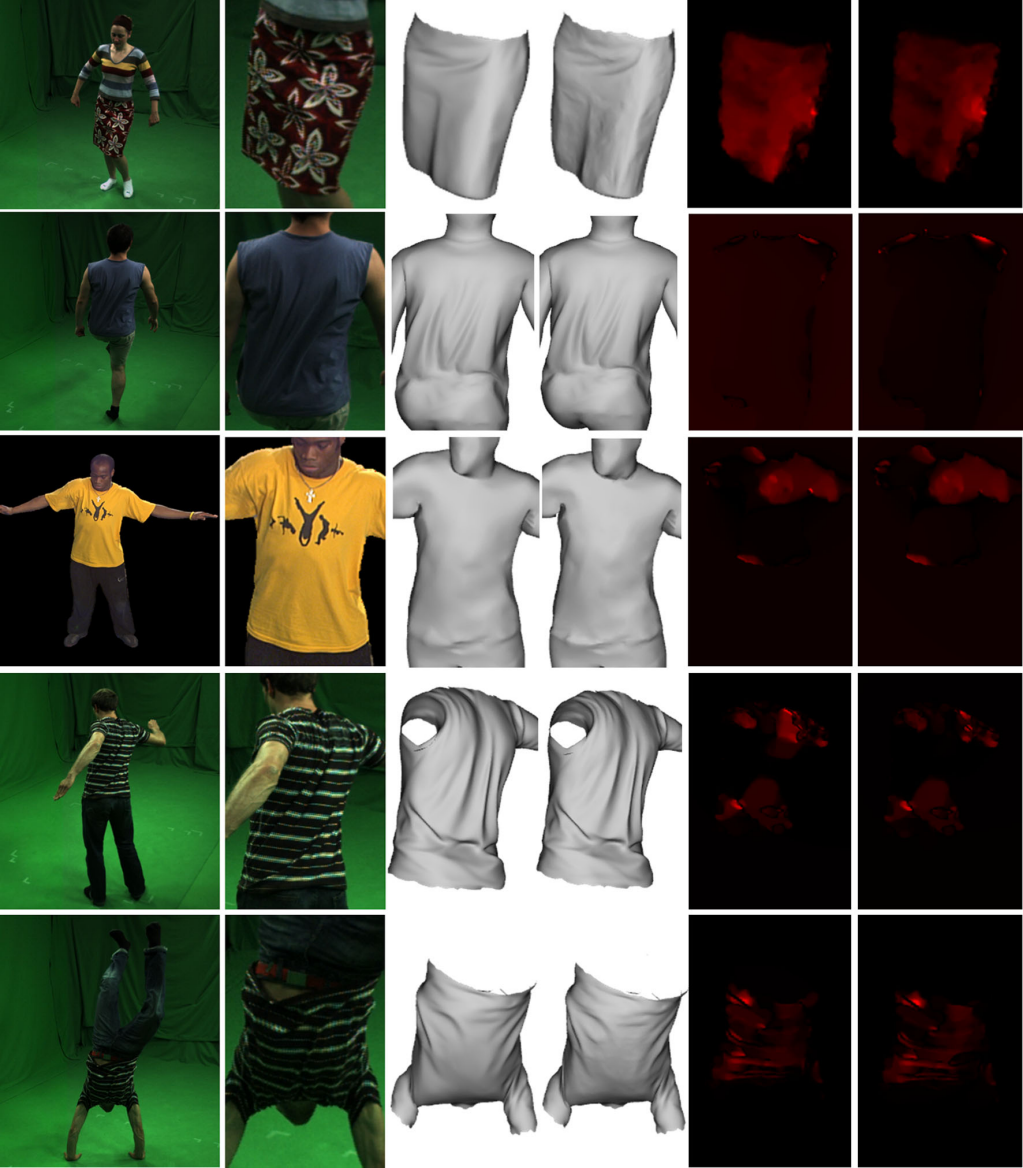
Results of our refinement approach for (from *top to bottom*) the *skirt*, *dance*, *pop2lock*, *wheel* and *handstand* animation sequences from an unused camera. From *left to right* input image, input image zoom-in, rendered input mesh, rendered output refined mesh, flow magnitude for the reprojected input mesh and the reprojected output mesh against the original input image. The refined meshes significantly decrease the flow displacement of the input sequence. Our method captures finer-scale details on highly textured surfaces , e.g. for the *skirt*, *wheel* and *handstand* sequences

**Fig. 11:**
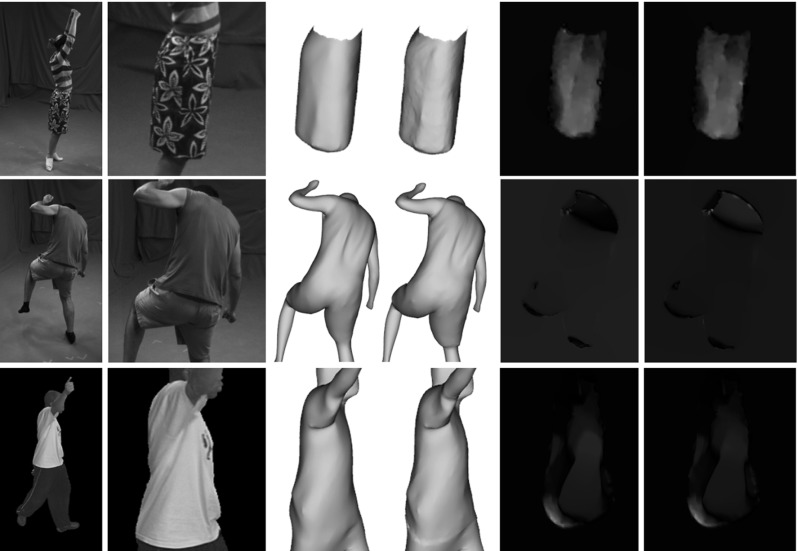
Results of our refinement approach for the smooth *skirt* (*top*), *dance* (*middle*) and *pop2lock* (*bottom*) animation sequences from an unused camera. From *left to right* input image, input image zoom-in, rendered input mesh, rendered output refined mesh, flow magnitude for the reprojected input mesh and the reprojected output mesh against the original input image. Our approach improves the quality of the input meshes by capturing additional surface details, visible in the zoomed-in version of each sequence

The *skirt* sequence is the most suited for our multi-view surface refinement technique thanks to its highly textured regions and accurate alignment with respect to the images. We focus on the refinement of the skirt surface region, which is highly textured. Textures lead to high-color variance of the Surface Gaussians, which in turn allow to estimate finer scale details and more accurate alignment, even in the inner parts away from the projected borders. As shown in Figs. 10 and 11, our method is able to capture additional fine-scale details for both the *skirt* and the smoothed *skirt* sequences using the default parameter settings depicted in Table 1. The incorporated details are best visible in the smoothed sequence version. The accompanying video shows some effects of the dynamics of the skirt being captured as well. See also the quantitative evaluation graphs in Fig. 7a, b, which report a consistent decrease in the flow error with respect to the input sequence. The deterioration in performance that happens around frame 400 is due to a temporal misalignment between the input skirt and the images, caused by an improperly captured full body rotation of the actor, that in turn voids the refinement attempts of the skirt surface. The alignment of the input sequence is stabilized in the following frames, allowing for correct refinement of the skirt motion. Although the original sequence presents highly accurate reprojection border alignment to the ground truth silhouettes, our quantitative evaluation shows that our approach captures the silhouette borders with even increased precision, particularly visible in the smoothed skirt sequence plots Fig. 8a.

#### *Dance* Sequence

The *dance* sequence shows strong deformations on the subject’s clothing during the jumping motions. We successfully refine the input geometry to include additional cloth dynamics, e.g. floating shirt, visible in the accompanying video, and quantitatively proven by the flow error plots in Fig. 7c, d. As discussed, inner details are only marginally captured when a shading effect appears, due to lack of textures. The overlap between the reprojected refined mesh onto the image space and the input image silhouette are effectively improved by our optimization approach, also reflected in the quantitative evaluation in Figs. 8b and 9. For both the original and the smoothed animation we used the default parameters setting in Table 1.

#### *Pop*2*lock* Sequence

The *pop2lock* sequence (Starck and Hilton 2007b) is challenging to refine with our approach due to the large homogeneously colored regions on the subject’s shirt. On top of that, the input mesh presents severe misalignments with respect to the input multi-view images with several time-consistency artifacts throughout the video. Nevertheless, using the default parameters setting in Table 1 we quantitatively report a reduced average flow error displacement with respect to the input mesh animation, both for the original *pop2lock* and the smoothed version, see Fig. 7e, f. Our refinement approach is unable for this sequence to improve the silhouette overlap consistently due to the strong shirt deformations accompanied by complex folds forming during the motion, see Fig. 8c. The visible picks in the silhouette improvement graphs correspond to those time intervals where the shirt has the least deformations, because the actor stands upright (visible in the video). In those few frames our refinement approach performs adequately, improving the original alignment, especially for the smoothed input sequence. The overlap could be further improved by switching off our regularization term, allowing for more freedom in the surface displacements, at a cost of less smoothed surface areas.

#### *Handstand* and *Wheel* Sequences

Both the *handstand* and the *wheel* sequences contain textured regions represented by the stripes in the shirt, which help in improving the surface details by coherently guiding the Surface Gaussian along the shirt deformation. However the *handstand* sequence is particularly challenging for our refinement approach, since the shirt is strongly deformed during the performance, especially in the upside-down pose after frame 50. On the other hand the *wheel* represents an example result of fast motion where the underlying geometry is temporally misplaced around frame 50, resulting in false underlying colors and therefore inaccurate refinement, see Fig. 7g, h. Our quantitative evaluation shows for both sequences an improvement in the flow displacement as well as improved silhouette boundaries (see the plots in Fig. 8d), except in the described challenging time intervals, where the input geometry and colors are temporally misaligned with respect to the images. As shown in the optical flow and silhouette improvement plots, our approach successfully recovers after frame 60.

**Fig. 12:**
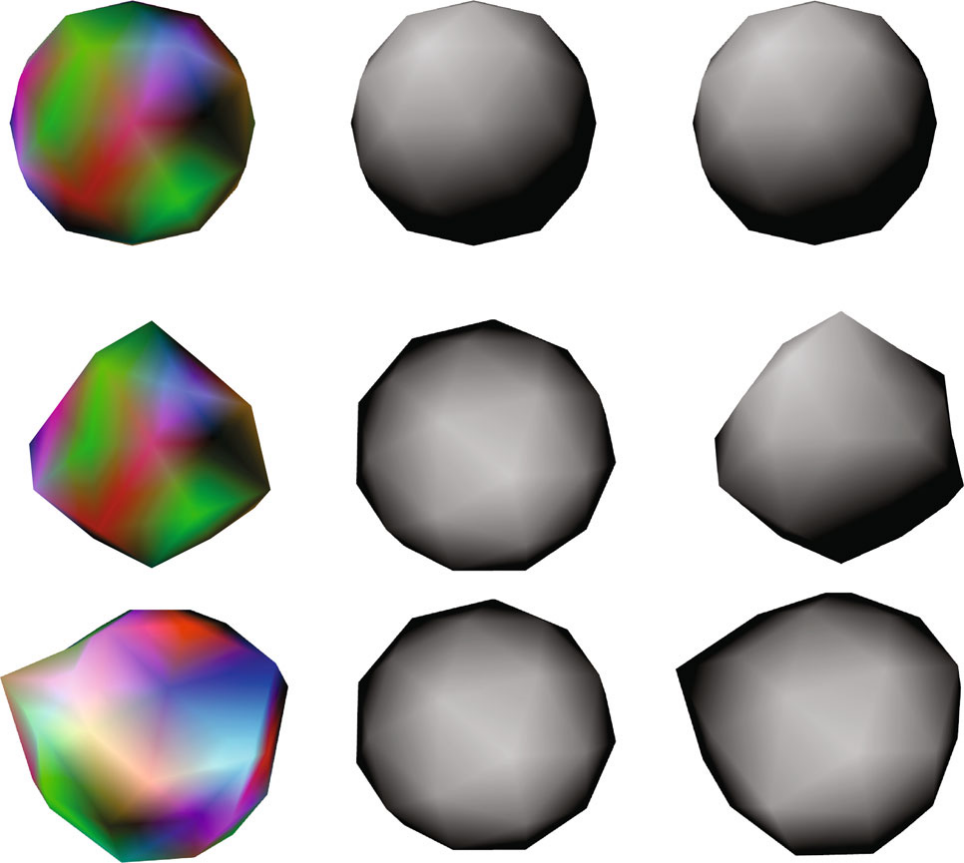
Results of our refinement approach for the synthetic sequences. From *top to bottom* no additional displacement, normal displacement and random displacement. From *left to right* one of the input multi-view images (out of 10), initial mesh and refined mesh. For generating these results we used the default parameter settings for all the sequences, except $T_{dist} = 90$ for all the sequences and $w_{reg} = 0$ for the *middle and bottom sequence*, aiming at capturing the simulated random displacement. For each sequence (from *top to bottom*) we obtained an average displacement error in $[\%]$ relative to the total volume size equal to 0.22, 1.84 and 7.1

**Fig. 13:**
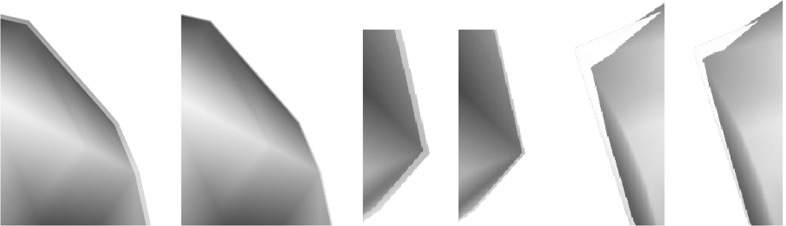
Effects of adding a displacement along the normal of the refined meshes equal to the standard deviation of the Surface Gaussians. The Figure shows the zoomed-in overlap between the input (semi-transparent) and resulting (*colored*) mesh of the synthetic sequence first without and then with additional displacement. From *left to right* overlap from the static sequence, from the sequence displaced along the normal and the sequence with random displacement. As shown by the figures, the increase along the normal produces better aligned results. The average errors in $[\%]$ relative to the total volume size for each refined mesh are in the order 0.22, 1.84 and 7.10, against the unchanged 0.69, 1.95 and 7.13 (Color figure online)

**Fig. 14:**
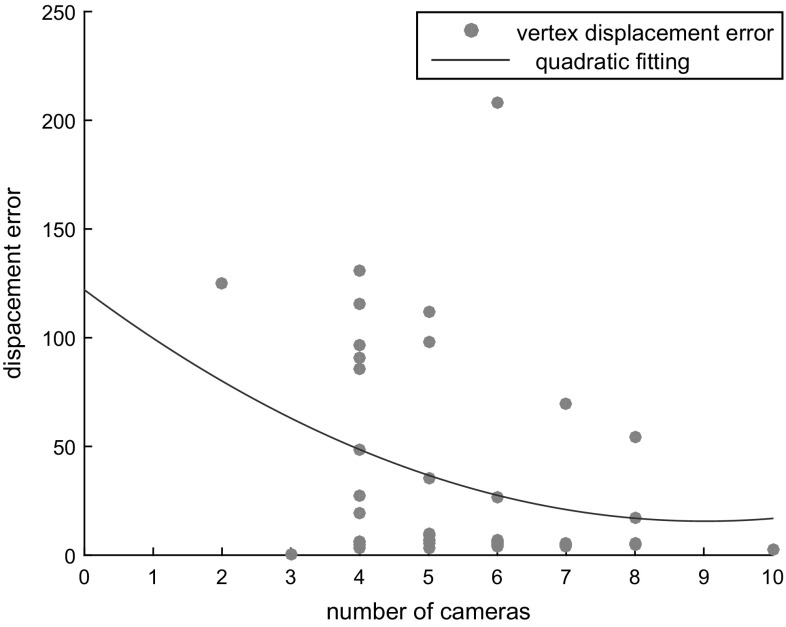
Average vertex displacement error when using different number of cameras. The error is computed on the *synthetic sphere* sequence by varying the number of input cameras

#### *Synthetic* Sequence

We additionally evaluate the performances of our approach on a *synthetic* sequence. Figure 12 shows the input and output mesh for each scenario. For the first unchanged scenario we use the default parameter settings from Table 1, while for the remaining two we used $w_{reg} = 0$ aiming at capturing the randomly generated displacements. For all scenarios we have used $T_{dist} = 90$ pixels to capture also larger displacements. Since we dispone of the ground-truth for this sequence, we compute exact average Euclidean displacement error, by summing up the vertex displacement norm from the ground truth divided by the number of vertices. The average error is then estimate as a percentage of the total object volume size. Even with large distortions (i.e. severe displacement) applied to the original geometries, our method is able to recover the fine geometric details with average errors lower than ${\le }7\%$ of the volume size. We further validate the additional $\epsilon$ we add to the optimized displacements of the Surface Gaussian (see Sect. 5.4), showing a quantitative improvement of the reconstructions, see Fig. 13. As we show in Fig. 14 the error increases when reducing the amount of views. This finding suggests that without providing more advanced surface smoothness term to handle missing depth, our approach is impractical for settings with few-cameras.

### Parameters

**Fig. 15:**
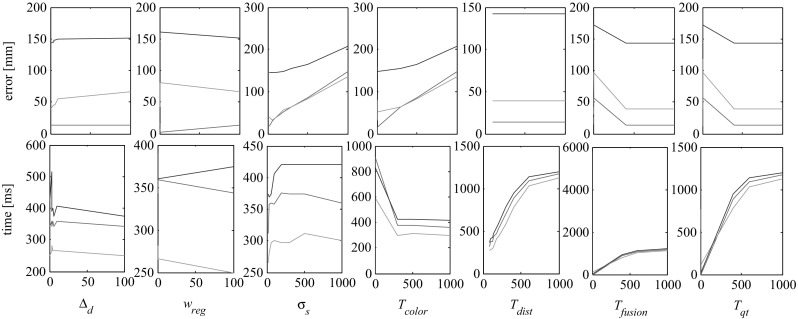
Influence of the parameters of the energy function $\mathbf E$ on the reconstructed error and computational time, in the *syntetic* dataset. The reconstruction error is estimated as average Euclidean displacement error from the given ground-truth in three different scenarios: input and target meshes are equal (no displacement, *red lines*), the target has a random displacement along the corresponding normal (normal displacement, *green lines*), the target has random displacement which may deviate from a normal displacement (random displacement, *blue lines*) (Color figure online)

In order to test the influence of the parameters used in our energy formulation, we run several experiments based on the *synthetic* dataset described at the end of Sect. 6.1. For each user-defined parameter, see Table 1, we change the parameter value within its pre-defined interval, while keeping the remaining parameters fixed, and run our refinement approach with this setting. The set of plots in Fig. 15 show the error and time performances obtained by varying each parameter.

As expected increasing $w_{reg}$ improves the static results, deteriorating the randomly displaced ones. Since we do assume a certain amount of smoothness in our scenarios, we have chosen a proper default regularization finding the most suited trade-off between smoothness and amount of captured details.

By increasing the standard deviation $\sigma _s$ size, we obtain slightly worse reconstructions. We believe this is due to the fact that the Gaussian color is taken from the corresponding vertex color of the ground-truth mesh, and not from the underlying pixel average, as we do for all the remaining sequences. In this case, the underlying pixel color poorly matches the Surface Gaussian color, leading to displacement errors.

The color similarity threshold $T_{color}$ restricts the evaluation of our similarity function w.r.t. a Surface Gaussian to a subset of Image Gaussians with similar color properties. The larger the threshold the more Image Gaussians are included in the evaluation, leading to an increasing computational time. The distance threshold $T_{dist}$ value highly depends on the sequence and mesh scale. Further increasing its value beyond its default ($T_{dist} = 90$ pixels for the *synthetic* sequence) does not improve qualitatively the reconstruction, instead it slows down the performances. On the other hand the fusion threshold $T_{fusion}$ and the quad-tree depth threshold $T_{qt}$ only produce accurate reconstruction results for high values, which in turn decrease the time performances. The smoothing weight $w_{temp}$ improves the stability of the refinement in time, without further improving the quality of the resulting surface. We show the effects of varying smoothing weight in the accompanying video.

### Limitations and Future Work

In this subsection we state the main limitations of our approach and provide possible solutions to be considered as a future work. We assume the input mesh sequence to be sufficiently accurate, such that smaller details can be easily and correctly captured by simply displacing vertices along their corresponding vertex normals. In cases where the input reconstructed meshes present misalignments with respect to the images (e.g. *pop2lock*) or if it is necessary to reconstruct stronger deformations, then our method is unable to perform adequately. Our refinement might be reformulated allowing more complex displacements, e.g. without any normal constraint. However such weaker prior on vertices motion requires more complex regularization formulation in order to maintain smooth surface, also to handle unwanted self-intersections and collapsing vertices. On top of that the increased number of parameters to optimize for (i.e. 3 times more, when optimizing for all 3 vertices dimensions, *x*, *y* and *z*) would spoil computational efficiency and raise the probability of getting stuck in local maxima solutions. The risk of returning local maxima solutions is still high when employing local solvers (e.g. gradient ascent) on non-convex problems as in our case. A possible solution is to use more advanced solvers, e.g. global solvers, when computational efficiency is not a requirement.

As we demonstrated in this Section, our approach is unable to densely refine plain colored surfaces with few texture (e.g. *pop2lock* and *dance*). A solution here is to employ a more complex color model that takes into account e.g. illumination and shading effects, at the cost of increased computational expenses.

We currently assign colors to the Surface Gaussians based on the underlying pixel average as seen from the best camera view. Among the available frames, we chose one of those where the input mesh is closely aligned to the input multi-view images in order to guarantee best color assignment. Apart from selecting the best frame, a way to improve the color assignment to the Surface Gaussian, taking into account the current parameters setting, is by solving our energy $\mathbf E$ w.r.t. the colors of the Surface Gaussians, instead of averaging underlying pixels. By doing so, the obtained colors are best suited for the current setting as they take into account the actual Image Gaussians size, color and position that are later used for optimization.

The used temporal smoothing term analytically formulates smoothing in time based on a window of 3 frames, that might be insufficient to fully eliminate time inconsistencies, e.g. for videos captured at high frame rates. We would like to further investigate into the impact of a larger window size on the overall time consistency of the reconstruction. On top of that our temporal smoothing approach only keeps the optimized displacements smooth in time and cannot correct time inconsistencies in the original input geometry. This limitation is particularly visible in the sequence *pop*2*lock*.

## Conclusions

Extending the original work presented in Robertini et al. (2014), we have presented an effective framework for performance capture of deforming meshes with fine-scale time-varying surface detail from multi-view video recordings. Our approach incorporates to the input coarse, over-smooth, meshes the fine-scale deformation present in the original video frames by deforming the input geometry to maximize photo-consistency on all vertex positions. We proposed a new model-to-image consistency energy function that uses an implicit representation of the deformable mesh using a collection of Gaussians for the surface and a set of Gaussians for the input images. The proposed formulation enables a smooth closed-form energy with implicit occlusion handling and analytic derivatives. We have extended the original optimization energy presented in Robertini et al. (2014) by adding an additional temporal smooth term that helps to avoid jittering and temporal inconsistencies in the output sequences.

We qualitatively and quantitatively evaluated our refinement strategy on 5 input sequences, initially obtained using 2 different state-of-the-art 3D reconstruction approaches: a template-free method, and template-based method. We demonstrate that in both cases the proposed method successfully recovers true fine-scale detailed geometry. Additionally, we have also shown the performance of our method on synthetic data, which we manually modeled and smoothed to create the ground truth. Results demonstrate that we successfully refine the distorted geometry to recover the original 3D model.

## Electronic supplementary material

Below is the link to the electronic supplementary material.

Supplementary material 1 (mp4 73471 KB)
